# Supplementary material for: Splice-Junction-Based Mapping of Alternative Isoforms in the Human Proteome
Source: Cell Rep. Author manuscript; Available in PMC 2020 Jan 15. (PMC6961840; doi:10.1016/j.celrep.2019.11.026)
Supplement: 2 [file NIHMS1546469-supplement-2.zip › DF1/PXD010154/Lung/Lung_23_SYPL1_ETSLHSPSNTSAPHSQGGIPPPTGL.pdf]

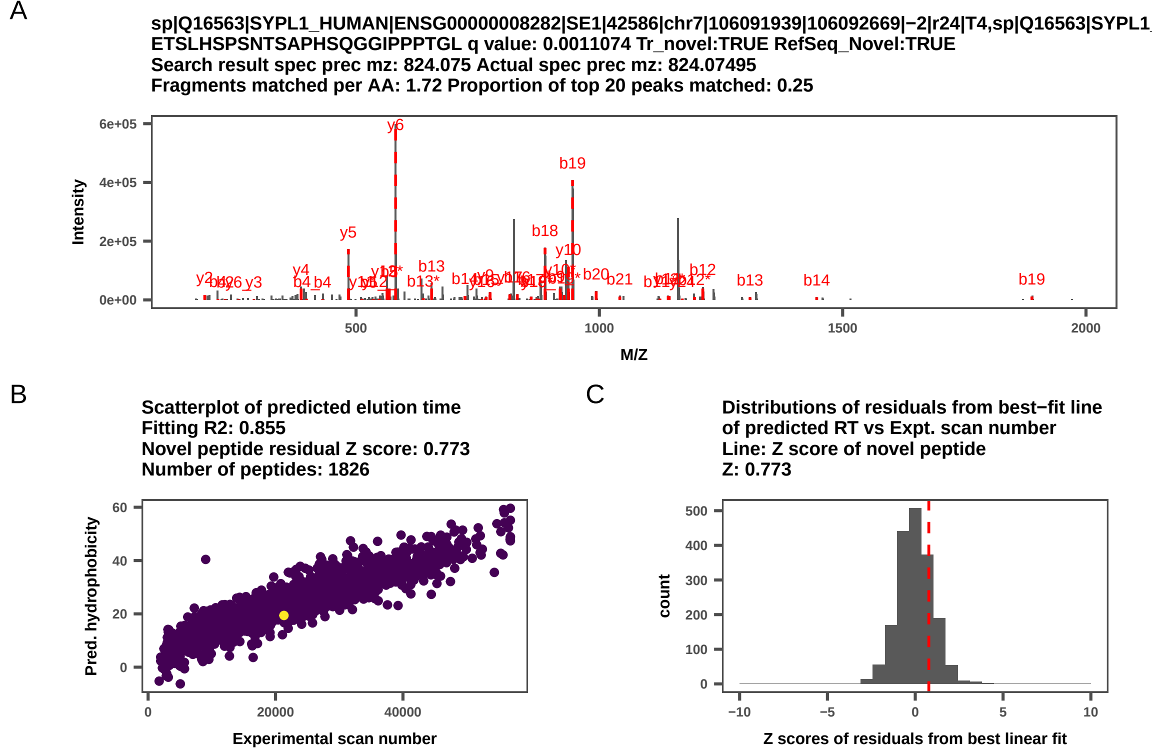
A
sp|Q16563|SYPL1_HUMAN|ENSG00000008282|SE1|42586|chr7|106091939|106092669|−2|r24|T4,sp|Q16563|SYPL1_
ETSLHSPSNTSAPHSQGGIPPPTGL q value: 0.0011074 Tr_novel:TRUE RefSeq_Novel:TRUE
Search result spec prec mz: 824.075 Actual spec prec mz: 824.07495
Fragments matched per AA: 1.72 Proportion of top 20 peaks matched: 0.25
Intensity
6e+05
4e+05
2e+05
0e+00
y6
b19
y5
b18
y10
b13
b13*
b12
b20
b21
b13
b14
b19
500
1000
1500
2000
M/Z
B
Scatterplot of predicted elution time
Fitting R2: 0.855
Novel peptide residual Z score: 0.773
Number of peptides: 1826
Pred. hydrophobicity
60
40
20
0
0
20000
40000
Experimental scan number
C
Distributions of residuals from best−fit line
of predicted RT vs Expt. scan number
Line: Z score of novel peptide
Z: 0.773
count
500
400
300
200
100
0
−10
−5
0
5
10
Z scores of residuals from best linear fit
